# Advances in Cellulose-Based Hydrogels for Drug Delivery: Preparation, Modification and Challenges

**DOI:** 10.3390/gels11120938

**Published:** 2025-11-21

**Authors:** Jiaxuan Di, Junge Li, Chao Sun, Longbin Xu, Xinyu Li

**Affiliations:** 1 Department of Polymer Materials & Engineering, College of Engineering, Yanbian University, Yanji 133002, China; 1234023183@ybu.edu.cn (J.D.); 1224021011@ybu.edu.cn (J.L.); 2 Department of Physics, Jilin University, Changchun 130012, China; sunc344@jlu.edu.cn; 3 Department of Materials and Chemical Engineering, College of Engineering, Yanbian University, Yanji 133002, China; 4 Department of Chemistry, College of Science, Yanbian University, Yanji 133002, China

**Keywords:** cellulose-based hydrogels, preparation methods, functional modification, drug delivery

## Abstract

A common and challenging issue in drug delivery is the premature release of drugs, which prevents them from reaching the target site. Finding suitable delivery materials has become a major research focus in the medical field. Cellulose-based hydrogels are a type of material with a three-dimensional network structure and good biocompatibility, offering significant advantages for drug delivery. This review begins with the raw materials of cellulose-based hydrogels and reviews their preparation methods and principles—including physical, chemical, and other special approaches—along with chemical modification strategies and their applications in medical drug delivery, such as drug carriers, drug release wound dressings, and so on. Special emphasis is placed on modification strategies to overcome the limitations of hydrogels, such as poor pH responsiveness, self-healing ability, and temperature sensitivity. It can be achieved by modifying the chemical chain itself, adding functional fillers, and constructing a dual network. Finally, the prospects of cellulose-based hydrogels in medical applications are discussed. Cellulose-based hydrogels, as drug delivery materials, are highly effective in biomedical applications and demonstrate significant potential for clinical translation in the field of precise drug release.

## 1. Introduction

In the field of medicine, the core of drug delivery lies in precisely controlling the transport and release of drugs within the body to improve therapeutic outcomes and reduce side effects [1]. Traditional delivery materials and methods face serious challenges in clinical applications including low targeting efficiency, lack of specific recognition, uncontrollable release behavior, and difficulty in crossing complex physiological barriers [2,3]. These issues greatly limit the clinical use of many effective drugs.

To address these challenges, hydrogels have gained widespread attention as advanced biomaterials [4]. Hydrogels are hydrophilic polymers that can absorb large amounts of water while maintaining a three-dimensional network structure [5]. They have tunable mechanical properties and excellent biocompatibility [6]. Through careful design, hydrogels can respond to specific pathological signals in the body—such as the acidic pH of tumor microenvironments or high enzyme activity—enabling intelligent, on-demand drug release and offering new possibilities for treatment.

Cellulose, the most abundant renewable organic polymer on Earth, is composed of linearly connected D-glucose units linked by β (1→4) glycosidic bonds. This regular molecular structure facilitates the formation of highly crystalline microfibrils through intra- and intermolecular hydrogen bonding, endowing cellulose with remarkable mechanical strength and chemical stability. The source and production method of cellulose ultimately determine its application. Key preparation methods include: (1) Microfibrillated cellulose (MFC) and cellulose nanocrystals (CNCs), obtained from wood or plant pulp via acid hydrolysis or mechanical treatment. CNCs, in particular, are known for their high modulus and unique optical properties due to their high crystallinity [7]. (2) Bacterial cellulose (BC), produced through microbial fermentation (e.g., by *Komagataeibacter xylinus*). BC is distinguished by its high purity, water-holding capacity, exceptional mechanical strength, and in situ molding capability [8].

Among the various hydrogels, cellulose-based hydrogels show great potential due to their excellent clinical compatibility. Cellulose possesses abundant hydrophilic hydroxyl groups, which grant it excellent hydrophilicity and water retention capacity, forming the basis for constructing hydrogel networks. More importantly, these functional groups serve as sites for chemical modification. Through reactions such as esterification, etherification, or oxidation, specific functional groups (e.g., carboxyl groups) can be introduced to enhance their solubility, reactivity, or biocompatibility. Simultaneously, cellulose exhibits outstanding mechanical properties, particularly in the case of nanocellulose (CNCs and BC). The nanoscale fibrous structures of these materials can form robust reinforcing networks within the hydrogel matrix, significantly improving the strength, toughness, and fatigue resistance of the hydrogels. This effectively overcomes the mechanical limitations often associated with traditional synthetic hydrogels. Furthermore, cellulose demonstrates remarkable biocompatibility and degradability. Its degradation products are non-toxic to the human body. Through appropriate chemical modifications, its biodegradation rate can be precisely tailored, making it suitable for biomedical applications ranging from short-term to medium-term use. These properties make cellulose-based hydrogels ideal candidates for advanced drug delivery strategies, such as localized and targeted therapy [7]. This review will summarize recent developments in the synthesis methods, chemical modifications, and applications of cellulose-based hydrogels in drug delivery and discuss future directions.

## 2. Fabrication of Cellulose-Based Hydrogels

The preparation methods for cellulose-based hydrogels are diverse, as shown in Table 1, primarily including physical cross-linking, chemical cross-linking, and other methods. Chemical cross-linking often yields hydrogels with better mechanical properties and stability [9]. Each method has unique advantages: physically cross-linked hydrogels are reversible and environmentally responsive, while chemically cross-linked ones exhibit strong mechanical properties and good heat resistance. Traditional hydrogel materials face limitations such as low mechanical strength, causing rupture, drying, or excessive swelling due to hydrophilicity, poor surface adhesion requiring auxiliary fixation, and complex preparation hindering large-scale production. Cellulose-based hydrogels can effectively overcome these drawbacks, enabling a greener and more efficient preparation process.

### 2.1. Physical Cross-Linking

Physical cross-linking forms a 3D network through reversible non-covalent interactions (hydrogen bonds, ionic bonds, electrostatic forces) between polymer chains like cellulose [24]. The main preparation methods are shown in Table 2. It avoids adding cross-linkers, simplifies production, prevents potential toxicity, and aligns well with cellulose’s hydroxyl-rich structure, which readily forms hydrogen bond networks. This results in good biocompatibility and promising prospects for drug delivery.

#### 2.1.1. Hydrogen Bond Dynamic Cross-Linking

**Figure 1:**
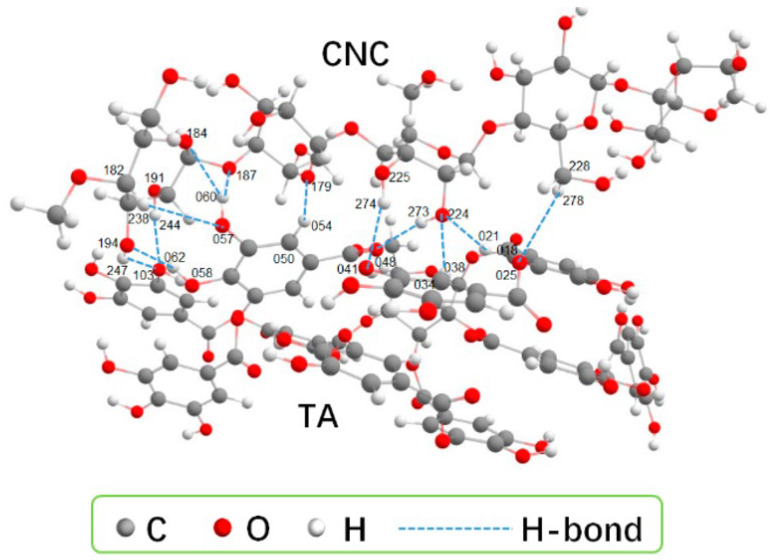
The optimized configurations for CNC-TA. Each TA molecule forms 12 H-bond interactions with CNC. Reproduced with permission from Ref. [25]. Copyright 2023, MDPI.

Traditional chemical cross-linking can lead to high initial drug release and poor biocompatibility due to cross-linker toxicity and irreversible networks [10,24,30]. Hydrogen bond dynamic cross-linking uses reversible hydrogen bonds between cellulose chains to create a 3D network, offering both safety and smart responsiveness. In 2018, Ye et al. [10] proposed a synergistic strategy that involved “pre-stretching and hybrid cross-linking”. They dissolved cotton liner pulp in an alkali/urea aqueous solution. This induced the internal hydrogen bonds of cellulose to align and fix along the force field direction. They successfully prepared various anisotropic cellulose hydrogels, obtaining purely hydrogen-bond cross-linked hydrogels. Lin et al. [25] used tannic acid (TA) as a gelling agent. As shown in Figure 1, when mixing the suspensions of CNCs (4.0 wt%) with TA (CNC:TA = 1:2), multiple hydrogen bonds formed between TA and cellulose nanocrystals (CNCs). The strong hydrogen bonding interactions significantly increased the viscosity of the CNC suspension and promoted its gelation. Liu et al. [30] introduced TA as a hydrogen bond enhancer into their system. The catechol groups of TA formed multiple hydrogen bonding cross-links with cellulose. The compressive strength of the resulting hydrogel increased sixfold compared to pure carboxymethyl cellulose (CMC). In 2024, Zhang et al. [31] first proposed a strategy to construct multiple hydrogen bonds. They dissolved softwood pulp cellulose directly in a salt solution containing Zn^2+^ and Al^3+^. Then, they copolymerized it with acrylic acid and acrylamide. This allowed hydrogen bonds in the hydrogel to break and reconnect, forming a relatively stable hydrogel (Ion-C-P(AA-co-AAm)). As a result, the improved anti-swelling ability (88.03%) and compressive performance (24.11 MPa) of the resultant were achieved and could maintain strain sensitivity and stability under extreme conditions. Recently, Zhang et al. [32] developed a method using charge-assisted hydrogen bonds. A strong hydrogen bond network was formed by the combination of the phosphate groups in DNA with the hydroxyl groups on the cellulose framework [11]. These cellulose-DNA hydrogels exhibited a specific surface area of 145.9 m^2^/g, compressive strength of 1.12 MPa, and laccase loading capacity of >937.3 mg/g. It also opened new possibilities for combining cellulose-based hydrogels as drug carriers with biological DNA. Although hydrogen-bonded hydrogels offer high biosafety and some intelligent responses, they still face challenges. These include insufficient mechanical strength and the susceptibility of hydrogen bonds to interference in physiological environments. Future work may focus on reinforcing with nanocellulose or creating hybrid networks with dynamic covalent bonds to improve hydrogen bond stability.

#### 2.1.2. Ionic Dynamic Cross-Linking Method

Ionic cross-linking is one of the most common mechanisms in self-assembled hydrogels. This method does not require toxic chemical cross-linkers. It uses dynamic coordination between multivalent metal (Fe^3+^, Ca^2+^/Mg^2+^, Zn^2+^) ions and anionic groups on cellulose derivatives. The ionic interactions form the 3D network structure of the hydrogel [26]. This builds a drug delivery carrier that combines green processing, stimulus responsiveness, and targeted controlled release capability. Dong et al. [26] added metal ion salts to an aqueous suspension of carboxylated CNF. The cations neutralized the negative charges on the CNF surface, rapidly initiating gelation and forming an interconnected porous nanofibril network. Similarly, gels can be obtained by screening the repulsive charges on nanofibrils via cation–carboxylate interactions. Shahriari-Khalaji et al. [27] increased the ultimate tensile stress of the gels to 0.29 plus or minus 0.06 by immersing BNC/Alg-Na in copper sulfate solution and conducting magnetic stirring at room temperature for 24 h, followed by washing with deionized water. This significantly exceeded the original stress. Recently, Yang et al. [12] used a bacterial cellulose (BC) membrane as a robust skeleton and introduced a dual-ion system of Zn^2+^ and Cu^2+^. Zn^2+^ formed the primary cross-linked network with carboxyl groups while Cu^2+^ acted as a secondary dynamic bond. They also introduced formate anions to regulate the aggregation state of polymer chains. This strategy enabled the hydrogel to achieve an ultrahigh ionic conductivity of 105 ± 5 mS cm^–1^, alongside satisfying mechanical strength (0.78 MPa). Although ionically cross-linked hydrogels are dynamic, reversible, and free of chemical cross-linkers, their mechanical strength is relatively low. Their stability is also poor at high ion concentrations. Therefore, developing multi-ion synergistic systems to enhance performance has become a research hotspot in drug delivery.

#### 2.1.3. Electrostatic Interactions

Electrostatic interactions refer to forming a 3D network through electrostatic attraction between oppositely charged polyelectrolytes. Examples include anionic cellulose derivatives and cationic polymers/ions. This method avoids the use of toxic cross-linkers [13,28,29]. The manufacturing process is relatively simple. Deng et al. [28] designed a series of injectable in situ cross-linking hyaluronic acid/carboxymethyl cellulose-based hydrogels using disulfide bonds. After chemically modifying HA and CMC to introduce thiol groups, dissolved oxygen mediated the oxidation of thiol groups to form disulfide bonds. When the carboxyl groups of CMC and the carboxyl/hydroxyl groups of HA created electrostatic repulsion, the disulfide bonds provided covalent cross-linking points. This balanced the stability and reversibility of the network, resulting in a stable hydrogel. Zhang et al. [29] mixed a negatively charged TEMPO-oxidized cellulose nanofiber dispersion with a positively charged partially deacetylated chitin nanofiber (PDChNF) dispersion at room temperature. Relying on their electrostatic attraction, they self-assembled into a 3D network within one minute. No cross-linking agent was added during the entire process, forming a physically cross-linked hydrogel. Besides electrostatic self-assembly, electrostatic adsorption can also prepare hydrogels. Hu et al. [13] adsorbed the copper complex of gamma-aminobutyryl-L-histidyl-L-lysine (GHK-Cu) onto hydroxyapatite microspheres (HAPs) via electrostatic interactions. Then, they mixed the HAP with CMC, glycerol, and water to form a GHK-Cu@CMHA gel. The experiment proved that this hydrogel could release 25.3% of GHK-Cu within 24 h and remained stable at 34% after 48 h, demonstrating a stable and controlled release curve without burst release, acting as a novel injectable soft tissue filler. Electrostatic interactions build dynamic networks through directional charge attraction, show significant advantages in drug delivery, and are environmentally friendly. However, their drug loading capacity for neutral drugs is relatively poor. Mechanical properties and stability still need further optimization through the design of new polyelectrolytes.

### 2.2. Chemical Cross-Linking

Chemically cross-linked hydrogels usually have higher mechanical strength than physically cross-linked ones [33]. They are mainly obtained through covalent cross-linking by multifunctional molecules. The main methods are shown in Table 3. Compared to physical cross-linking, specific cross-linkers are generally required. Initiator residue toxicity can be high. Carboxyl and hydroxyl groups are the most commonly used active groups in cellulose and its derivatives for forming covalent bonds.

#### 2.2.1. Covalent Cross-Linking

Covalent cross-linking involves permanently connecting polymer chains through irreversible chemical bonds. Examples include ester bonds, ether bonds, and imine bonds. This strategy forms a three-dimensional network structure. Compared to physical cross-linking, hydrogels from this method are more stable. They have strong resistance to swelling and are more conducive to precisely controlled release. As early as 1928, German scientists discovered the Diels–Alder reaction [14], which involves cycloaddition between diene and dienophile compounds to form a six-membered ring. In 2011, Ax et al. [39] used this principle and first introduced furan rings onto hydroxyethyl cellulose (HEC) via esterification, obtaining furanated cellulose. The furan groups then underwent a D–A cycloaddition with *N*-bismaleimidehexane at 60–80 °C, forming a covalently cross-linked network. This network was thermoreversible. After 5 heating–cooling cycles, the gel strength retention rate was still >85%. In 2018, Pan et al. [34] used epichlorohydrin as a cross-linker. They pre-crosslinked sugarcane bagasse cellulose (SBC) with CMC under alkaline conditions. Then, they introduced *N*-isopropylacrylamide (NIPAM) for free radical polymerization, forming a purely covalently cross-linked interpenetrating network (IPN) hydrogel. In this hydrogel, SBC formed the main support structure, and CMC provided pH responsiveness. At 50% of CMC, the saturated swelling ratio reached 97.2 times. This gave the hydrogel great potential for controlled drug release. Recently, Dong et al. [35] successfully prepared a redox-responsive network gel. They used a selenium-bridged bicyclononane dihalide as the cross-linker. The Se-BCN-crosslinked material showed significantly greater tensile strength (by 80%) and elongation at break (by 250%). Its halogen groups reacted with the carboxyl groups of cellulose, generating covalent bonds and further forming the redox-responsive network. Although covalent bonds provide anti-swelling properties and high-temperature stability and offer stronger adaptability to strong acid/base environments, their reactions are irreversible and poorly recreatable. Cross-linker residue can cause high biotoxicity. Balancing the stability and reversibility of chemical bonds, intelligently regulating the network structure and reducing or even eliminating the use of toxic cross-linkers are major challenges researchers currently face [34,35,39].

#### 2.2.2. Schiff Base Reaction

**Figure 2:**
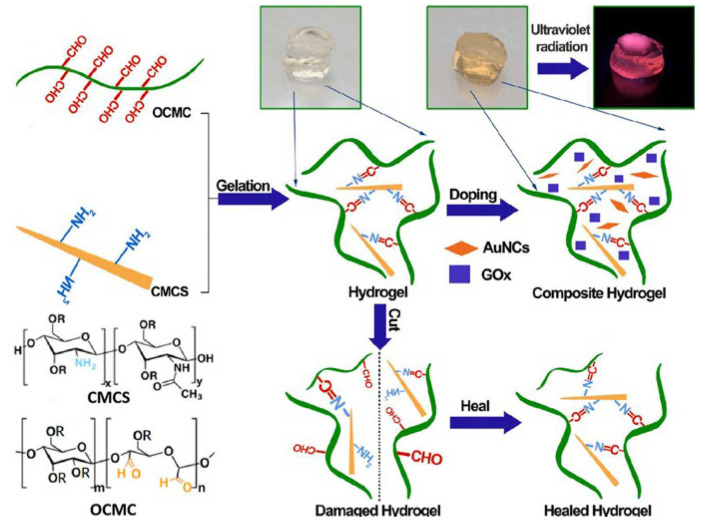
Schematic illustration for the gelation and self-healing of the AuNC-CMCS/OCMC hydrogel. Reproduced with permission from Ref. [15]. Copyright 2021, Elsevier.

The Schiff base reaction involves the condensation of an aldehyde or ketone with a primary or secondary amine under acidic or neutral conditions. This reaction generates a Schiff base. It can not only construct the 3D network structure of hydrogels but also introduce functional groups to expand their applications. It offers advantages like pH responsiveness, biocompatibility, and mild reaction conditions. In 2021, Zhang et al. [36] crosslinked the amino groups of oxamido cyclosiloxane (OCAPS) with the aldehyde groups of oxidized hydroxypropyl cellulose (HPC) via a Schiff base reaction and prepared a series of injectable nanocomposite hydrogels for the first time. Results showed that OCAPS was well-dispersed in the hydrogel. The self-healing rate was as high as 95%. In the same year, Shen et al. [15] performed a Schiff base reaction between the aldehyde groups of OCMC and the amino groups of CMCS that formed dynamic imine bonds. Simultaneously, hydrogen bonds formed between the carboxyl and hydroxyl groups, and they successfully constructed a CMCS/OCMC hydrogel. Then, just as in Figure 2, they encapsulated AuNCs and GOx in this hydrogel as fluorescent bioprobes, creating a special fluorescent hydrogel. This exhibited high sensitivity for glucose sensing with a linear detection range of 100 μM to 5 mM and a detection limit of 0.029 mM, which covered the level of glucose in clinical detection. Recently, Tohamy et al. [37] started from an environmental perspective and used recycled sugarcane bagasse (SC) as the raw material. Periodate oxidation produced dialdehyde cellulose (DAC). The aldehyde groups of DAC and the amino groups of chitosan formed stable imine bonds via the Schiff base reaction, building a 3D network. This reaction was accelerated by microwave assistance, achieving high-density covalent cross-linking. Experiments verified that its release rate reached 82% in simulated intestinal fluid (pH = 7.4), with an initial release rate < 15%. It is an excellent material for drug delivery. The role of the Schiff base reaction in hydrogel formation is to provide physical cross-linking points. Its dynamic reversibility and adaptability enhance self-healing ability. This self-healing property is crucial for extending the hydrogel lifespan and improving stability in complex environments. However, problems like complex synthesis processes and poor long-term stability remain. How to precisely control dynamic bonds in hydrogels, along with green synthesis and precise drug release, may be current research trends.

#### 2.2.3. Esterification Reaction

In preparing cellulose-based hydrogels, esterification refers to the reaction between the hydroxyl groups of cellulose and cross-linkers like acid anhydrides. This forms ester bonds, crosslinking cellulose molecules and converting them into hydrogels with a 3D network structure. Zhang et al. [16] utilized esterification to graft 3-aminophenylboronic acid onto carboxyethyl cellulose. Further crosslinking with polyvinyl alcohol via dynamic boronic ester bonds formed a gel. When the strain of the 1/4 hydrogel was increased from 5% to 100% under 6.28 rad/s, the G′ remained constant, indicating that not much cracking formed in the hydrogel. It possessed self-healing and injectable properties, used for controlled doxorubicin release. Liu et al. [38] designed an esterification reaction between the hydroxyl group of cabazitaxel (CTX) and the carboxyl group of CMC. This formed a CTX–CMC conjugate. Then, mPEG was linked to CMC via esterification. The hydrophobic CTX core and hydrophilic CMC-mPEG shell spontaneously formed CTX-CMC-mPEG polymer micelles in aqueous solution. The experimental results showed apoptotic rates of 45.2% for the CTX solution and 39.7% for the CTX-CMC-mPEG micelles and provides an efficient antitumor effect with reduced toxicity of CTX. Recently, Xu et al. [17] grafted 4-carboxyphenylboronic acid (4-CPBA) onto a gelatin skeleton via phenylboronic esterification. This created a pH-responsive dynamic covalent bond. They also introduced sulfonated cellulose nanocrystals to build oriented microchannels, obtaining a biomimetic hydrogel. The amino groups of gelatin condensed with the carboxyl group of 4-CPBA, forming an amide bond. The phenylboronic acid groups formed reversible boronic ester bonds with the catechol groups of sodium danshensu (DSS), achieving precise DSS release. The presence of ester bonds can directionally adjust the hydrogel’s mechanical strength, swelling properties, and biodegradability. The final cumulative DSS oxygen release was 2.24 times and 3.20 times that of the control group, respectively. However, issues like uneven substitution degree and reagent toxicity residue remain. How to make hydrogels greener and more intelligent is a significant challenge facing current research.

### 2.3. Other Cross-Linking Methods

Other cross-linking methods aim to avoid the irreversibility of physical methods and the toxicity of chemical cross-linkers. These mainly include biologically dynamic triggered cross-linking and photo-initiated cross-linking (Table 4). These methods offer stronger specificity and tailored preparation needs.

#### 2.3.1. Bio-Dynamic Cross-Linking

**Figure 3:**
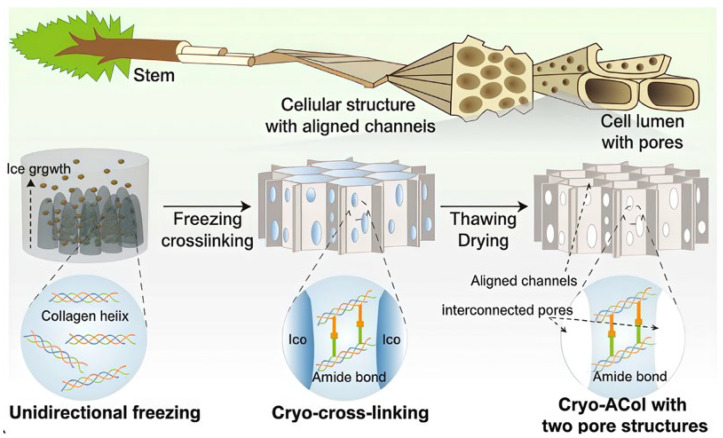
Inspired by the hierarchical structure of trees, a collagen dressing (Cryo-ACol) with ordered channels and interconnected pores was prepared. Reproduced with permission from Ref. [19]. Copyright 2025, Springer.

Bio-dynamic cross-linking refers to a process that mimics reaction mechanisms between biological molecules within a biomaterial system. It achieves reversible cross-linking to construct or regulate a 3D cross-linked network structure and possesses advantages like dynamic reversibility. It can be realized by mimicking the adaptive capacity of organisms, directly using biological molecules themselves, or simulating the extracellular matrix, among other methods. Inspired by pH changes during wound healing, Cheng et al. [18] developed a microenvironment-feedback-regulated hydrogel. This gel uses the initial alkaline pH of the wound as a “fuel” and the production of bio-catalyzed acid as an “anti-fuel”. An imine bond cross-linked network, formed from oxidized sodium alginate and gelatin, encapsulated glucose oxidase (GOx) and catalase (CAT) for treating diabetic wounds. It formed a self-feedback regulation loop based on in vivo enzyme release. Song et al. [19] were inspired by tree vessels and incorporated DNA immunomodulatory intelligence (Figure 3). Their team covalently anchored calf thymus DNA to a biomimetic directional macroporous collagen scaffold via EDC/NHS chemistry. This allowed DNA to act as both cross-linking points and actively enrich CD4+ T cells, promoting their secretion of anti-scarring factors while inhibiting the expression of the scarring marker α-SMA. They successfully developed a bifunctional collagen dressing combining exudate management and immune regulation for early burn management. Jia et al. [20] studied the mechanism of liquid–liquid phase separation in membraneless organelles and the formation of percolated molecular networks via multivalent interactions. They designed a system where multivalent specific interactions between proteins or peptides spontaneously form a stable mesoscopic percolation network throughout the entire biomolecular condensate during phase separation. This provides new ideas for synthetic hydrogels. The core of bioinspired cross-linking is to replicate both form and function, focusing on duplicating dynamic, self-adaptive, intelligent, responsive, and emergent functional properties. How to achieve multi-scale synergistic cross-linking and deep integration with living organisms has become a new research direction.

#### 2.3.2. Photo-Initiated Cross-Linking

Photo-initiated cross-linking uses light of a specific wavelength to activate a photoinitiator, generating free radicals. These radicals initiate monomer polymerization and promote the formation of stable chemical bonds between polymer chains, thereby building a three-dimensional network. Compared with traditional techniques, photo-initiated polymerization confines cross-linking to the irradiated area. This allows for the precise selection of cross-linking sites and offers advantages like mild conditions, rapid curing, and low cost. Researchers began intensive studies after first observing the photopolymerization of cinnamic acid as early as 1895. Hua et al. [21] constructed a double-network HPC hydrogel through dual mechanisms: photo-initiated free radical polymerization and photo-induced imine cross-linking. It achieved ultrafast gelation within one second; the compression stress of the HPC-Low and HPC-High gel was seven- and eightfold greater than that of the HANB/GL gel and 72- and 86-fold greater than that of the HAMA. It gave gel a high mechanical strength and considerable tissue adhesion. Recently, Li et al. [22] synthesized intelligent cellulose nanocrystal-based photonic hydrogels with enhanced mechanical properties and dynamic structural color by controlling the equilibrium time of CNC and AM precursors. They achieved long-range ordered structures, exhibiting high strength, toughness (undergoing twisting while being extended to >10 times their original length), elasticity, and dynamic optical response capability, becoming candidates for advanced sensing technologies. Wichai et al. [23] used a many-body dissipative particle dynamics model and prepared a CNC-NB/CMC-DA/TOB bio-adhesive via UV curing. This hydrogel exhibited high adhesion and bonding strength, rapidly gelling within thirty seconds under photocuring conditions. Although photo-initiated hydrogels have strong advantages in trauma repair and rapid intraoperative hemostasis, some photoinitiators still have cytotoxicity. Furthermore, light penetration through skin is limited. The metabolism after application and the adaptability of surrounding tissues still require deeper investigation.

In addition, methods like microbial fermentation-induced cross-linking, magnetic response, and photothermal driving are also research hotspots in the preparation of cellulose-based hydrogels.

## 3. Functional Modification of Hydrogels

The three-dimensional network structure and hydrophilicity of cellulose hydrogels allow them to absorb and retain large amounts of water without dissolving. This characteristic gives them unique advantages in controlled drug release [40]. However, unmodified cellulose hydrogels have problems like insufficient mechanical strength, low drug loading capacity, and a lack of intelligent responsiveness. These issues make it difficult to meet the demands of complex drug delivery scenarios. Therefore, researchers have introduced various functional fillers and constructed dual-network structures. They have successfully developed many new hydrogel systems with excellent mechanical properties, drug loading capacity, and stimulus responsiveness. These systems have tremendous potential in targeted therapy and controlled drug release. As shown in Table 5, modification methods and their limitations vary significantly for different target properties.

### 3.1. Self-Healing Performance of Hydrogels

Self-healing ability is an important property of hydrogels. It allows them to recover their original structure and properties after damage. This self-repair improves the efficiency and persistence of drug delivery. Most self-healing hydrogels are prepared based on the dynamic and reversible nature of dynamic chemical bonds. Introducing dynamic chemical bonds into the hydrogel network enables a reversible breakage-recombination reaction upon damage, restoring the original state. Due to this reversibility, self-healing hydrogels with dynamic bonds can undergo multiple repair cycles [41]. Whether using intrinsic materials like disulfide bonds and dynamic boronic ester bonds, introducing fillers like TA or metal ions, or constructing dual-network structures, the core goal remains the same, which is to introduce multiple dynamic reversible cross-linking points into the hydrogel network to enhance its self-healing capability.

**Figure 4:**
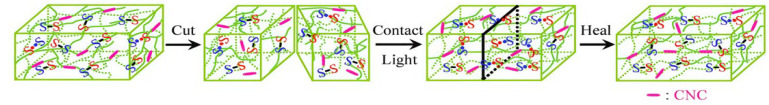
Schematic illustration of the self-healing process of CNC-containing gel. Under visible light irradiation, the content of CNC was 2.2% and the storage modulus was 6865 kPa. Reproduced with permission from Ref. [50]. Copyright 2018, MDPI.

Cellulose itself contains numerous surface hydroxyl groups. These groups can form intra and intermolecular hydrogen bonds, enhancing the hydrogel’s self-healing ability. Cellulose-based hydrogels inherently possess strong self-healing capacity. The CNC content in the system affects the repair efficiency [50]. Observation confirms that CNC presence accelerates self-healing to some extent. Wenyan Li et al. introduced cysteine into CNC, which regulated the cross-linking density of dithiocarbamate bonds in the system. The dithiocarbamate bonds in gels containing CNC can homolytically cleave under visible light, generating radicals. As shown in Figure 4, when fractured surfaces contact each other, radicals recombine on the newly contacted surfaces through exchange and transfer reactions. This reconstructs the disulfide bonds and achieves healing of the broken surfaces. The content of CNC in this gel is 2.2%, and it has excellent extensibility, capable of being stretched to nearly 42.6 times its original length. Yin et al. [51] used the Schiff base reaction between the aldehyde groups of oxidized microcrystalline cellulose and the amino groups of carboxymethyl chitosan (CMCS) to form a hydrogel. Its shortest clotting time was 54 s and demonstrated good self-healing ability and controlled drug release capability. Wang et al. [52] crosslinked polyvinyl alcohol (PVA) through dynamic boronic ester bonds. They combined it with hydrogen bonds from CMC using a one-pot method, without any catalyst. The resulting PVA/CMC hydrogel achieved a strain at break of up to 1910% at the rupture point after adhesion. It also rapidly self-healed within just 3 min.

Fillers, also known as additives, are solid materials added to a matrix during material preparation. They improve product performance, reduce cost, or impart new functions. In 2017, Zhang [53] chose Fe^3+^ as a filler for ionic coordination. Assisted by multiple hydrogen bonds, the resulting hydrogel exhibited excellent self-healing properties. In 2020, Yan et al. [54] introduced TA as a filler into CNC. They incorporated it into an ionic hydrogel system derived from various functional acrylic monomers. TA carries a large number of phenolic hydroxyl groups, which can form multiple hydrogen bonds. It also reacts with poly(ethyl methacrylate) to form stronger boronic ester bonds. This hydrogel could self-heal within 30 min without external stimulation. Recently, Gu et al. [42] linked PVA and CNC via hydrogen bonds using surface amino, carboxyl, and other active groups, obtaining a PBGTC hydrogel. They constructed a multi-crosslinked interpenetrating network system with dynamic covalent bonds and dual hydrogen bonds around the cellulose nanocrystals (CNCs). Experiments proved that the synergistic interaction between dynamic boronate bonds and dual hydrogen bonds gave the PBGTC hydrogel a remarkable self-healing function, with a self-healing rate as high as 93.3%.

Dual-network structures are evolving toward enhanced self-healing capabilities. Fu et al. [43] crosslinked chitosan derivatives with polyacrylamide (PAM) to form a dual network. The breakage and recombination of dynamic imine bonds and hydrogen bonds provided self-healing ability to this network. Due to the reversibility of imine bonds, the hydrogel could self-heal up to 84.2% under alkaline stimulation at 35 °C. Ma et al. [55] used a cross-linked network of SA and CS formed by electrostatic interactions as the second network. SA provided numerous hydroxyl and carboxyl groups as cross-linking sites, forming a dense hydrogen bond network. This resulted in excellent self-healing performance. Furthermore, Jiang et al. [56] utilized host–guest interactions between β-cyclodextrin (CD) and 1-adamantane (AD). They prepared an AD-CHO guest polymer via esterification, then obtained a dual-network hydrogel through molar mixing. Experiments showed that its mechanical properties were significantly improved and its self-healing efficiency was as high as 97.5%. It was also injectable, showing great potential in biomedical applications. Modified cellulose hydrogels have a prolonged service life and can serve as injectable carriers, reducing the need for invasive surgery. Their high self-healing ability gives them broad application prospects in the field of precision medicine.

### 3.2. pH Responsiveness of Hydrogels

The essence of pH responsiveness lies in the protonatable and deprotonatable groups within the material. These groups ionize under different pH environments. This changes the hydrogel’s network structure and intermolecular forces. Consequently, it causes the hydrogel to swell or shrink, achieving drug “release” or “storage [57,58,59]”. The surface of cellulose itself mainly has neutral hydroxyl groups and its response to pH changes is very weak. To solve this weakness, researchers mainly use three methods: modifying molecular chains, adding fillers, and constructing dual-network structures.

Modifying molecular chains is a fundamental method to address pH responsiveness. Researchers functionalize the molecular chains of cellulose by introducing pH-sensitive chemical groups. This directly changes the chemical properties of cellulose at the molecular level and significantly improves the intelligent response performance of the hydrogel, enabling precise pH regulation. Introducing carboxymethyl groups is the most common method. It contains abundant carboxyl groups, which ionize in alkaline environments, increasing the swelling degree. In 2014, Seki et al. [57] developed a hydrogel sensitive to pH changes. They used fumaric acid and malic acid at different concentrations, introducing carboxymethyl and hydroxyethyl groups. Research showed that the carboxyl groups in CMC ionize in alkaline environments. This generates electrostatic repulsion, causing hydrogel swelling. In acidic environments, they protonate and contract, which achieves pH responsiveness. Öztekin et al. [58] encapsulated curcumin (CR) with CMC. In the tumor microenvironment, the carboxyl groups in CMC are protonated. This reduces solubility and swelling capacity, aiding drug release in acidic environments. At neutral pH, the carboxyl groups in CMC are deprotonated, making them more hydrophilic and swollen. This allows for faster drug release in neutral or alkaline environments. In addition, grafting pH-sensitive polymers is a new approach. The high specific surface area and abundant functional groups of nanocellulose provide more sites for grafting pH-responsive groups. Pandey et al. [59] directly used acrylamide (Am) as a functional monomer and grafted it uniformly onto a BC skeleton via microwave irradiation. This introduced polymer chains rich in amide groups onto the molecular chains. Drug loading experiments proved that the drug release percentage was higher at pH = 7.4 compared to pH = 1.5. It showed no tissue cytotoxicity and qualified as a drug carrier. Similarly, Zhang et al. [44] grafted acrylic acid onto the ends of BC molecular chains. This enabled an intelligent response to wound pH and drug release. Experiments proved that after loading curcumin, the hydrogel showed good antibacterial activity against common wound pathogens like E. coli and S. aureus. The key advantage of modifying molecular chains is the precise design of the hydrogel’s response performance at the molecular level. However, the modification process may involve toxic reagents, increasing production costs. Safer and more reliable methods are needed for large-scale production.

Selectively adding certain functional fillers is a commonly used modification method. It can enhance mechanical properties and introduce additional pH response mechanisms. Way et al. [45] added carboxylic acids or amines as fillers to functionalize the CNC surface, giving it pH responsiveness. Further experiments showed that these pH-responsive CNCs could be incorporated into a polyvinyl acetate matrix. This produced mechanically adaptive, pH-responsive nanocomposite films. Akram et al. [60] used acrylic acid (AA) as a filler. They prepared a hydrogel matrix using three polymers: pectin, guar gum, and cellulose acetate phthalate (CAP). This enhanced the pH sensitivity and controlled release capabilities. When the pH was 7.4, the swelling rate reached 76.70%, and it was negligible at pH = 1.2. Because AA is mostly non-ionized in acidic environments, hydrogel swelling is limited. This minimizes premature drug release [61]. The optimized mixture of CAP, pectin, guar gum, and AA resulted in a hydrogel with enteric protection, pH sensitivity, and prolonged mucosal contact time, which ensured site-specific and sustained CT delivery. Zhang et al. [62] showed that using SBA-15 as a filler, through in situ graft polymerization of acrylic acid on the CMC skeleton, could successfully incorporate well-dispersed SBA-15 into a highly absorbent hydrogel. This improved the swelling capacity and swelling sensitivity. It could adsorb more drug molecules and release them under specific pH conditions, enhancing the pH-responsive drug release performance of the hydrogel. The core advantage of adding fillers is that they improve pH responsiveness while enhancing the overall performance of the hydrogel, such as mechanical properties and drug loading capacity. However, issues like uneven filler distribution or impacts on biocompatibility may occur. Therefore, optimizing the filler type and incorporation method is still needed to achieve the best results.

**Figure 5:**
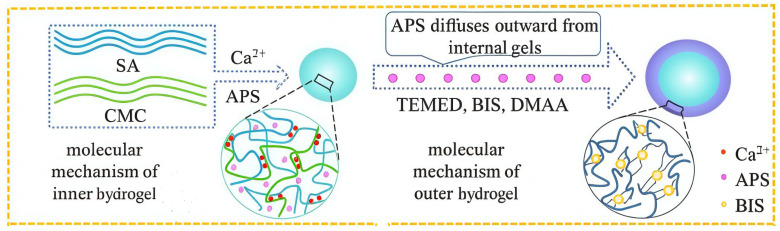
Preparation schematic diagram of a double-layer hydrogel. Reproduced with permission from Ref. [63]. Copyright 2021, Nature.

Constructing a dual-network structure involves redesigning the hydrogel framework at both the macro and micro structural levels. Hu et al. [63] utilized the diffusion method, as shown in Figure 5, and used natural polymers SA and CMC, cross-linked separately with Ca^2+^, to form an inner layer with an IPN structure. Due to the introduction of SA and carboxymethyl groups, this inner layer is more sensitive to pH in the weakly alkaline intestinal environment and avoids drug leakage in the stomach. Acrylamide and its derivatives were used to prepare the outer hydrogel layer via free radical polymerization [64]. It formed an outer coating on the SA-CMC surface, further preventing the inner hydrogel from swelling and the internal contents from diffusing. This ultimately achieved sustainable drug release. Drug release results showed the hydrogel was highly sensitive to pH, enabling site-specific release in vivo. Cui et al. [65] used polydopamine-coated CNC to construct physical and hydrogen bonds. They added CNC, polymerizable deep eutectic solvent (PDES), and anthocyanin to form a dual-network hydrogel. CNC enhanced the mechanical properties (2.95 and 69.57 times higher tensile strength and toughness than pure bioderived PDES) while PDES provided self-healing (>90%) and ionic conductivity. The anthocyanin coating provided pH responsiveness. Sweat pH detection experiments proved that the hydrogel had sensitive pH responsiveness, offering a new perspective for medical product development. Wang et al. [46] designed a fertilizer using CNF/SA as the first network and a metal–organic framework as the second network that had an excellent pH-responsive controlled release ability. This provides new ideas for research and development in the drug delivery design field. Dual-network structure design can regulate hydrogel properties from multiple dimensions. However, the manufacturing process is often complex, and reproducibility is hard to guarantee. The future may require more effort in designing novel structures. In summary, all three modification methods have pros and cons. Future work should consider green development, multiple responses, large-scale production, and cost control [46,63,64,65].

### 3.3. Temperature Responsiveness of Hydrogels

The essence of temperature response in hydrogels is their ability to swell and shrink according to temperature changes. This volume change facilitates the release of loaded drugs. However, cellulose itself lacks significant thermosensitivity. This limits its application effectiveness, making it difficult to respond sensitively to external temperature changes and achieve targeted drug delivery. Therefore, strategies like chemical modification, adding functional fillers, or constructing dual-network structures to enhance their temperature response have become key research directions for developing high-performance intelligent delivery systems.

Chemical modification involves chemically modifying the cellulose molecules themselves. This introduces thermosensitive groups or alters its microstructure, thereby improving its temperature response capability. The most commonly grafted thermosensitive polymers are poly(*N*-isopropylacrylamide) (PNIPAM) and poly(*N*-vinylcaprolactam) (PNVCL), which exhibit a lower critical solution temperature (LCST) around 32 °C in aqueous solution. Based on these temperature-sensitive polymers, nanogels swell at low temperatures and shrink at high temperatures. They can display a volume phase transition temperature (VPTT) close to physiological temperature, offering unique advantages in the medical field [47]. Compared to PNIPAM, PNVCL is non-toxic, has strong biocompatibility, and is hydrolytically stable, giving it a wider application range [66]. Yang et al. [67] used a hydrolyzed epoxidized soybean oil-grafted HEC polymer as an emulsifier and stabilized the dispersion of PNVCL in water. With an increase in the H-ESO-HEC content, the swelling capacity was improved from 16 g/g (Gel-1) to 23 g/g (Gel-4) at 20 °C. It also had different swelling ratios at different temperatures, exhibiting excellent temperature responsiveness. Additionally, Yi et al. [68] used a one-pot method to introduce CMC and hydroxypropyl cellulose (HPC) into a polyacrylamide (PAM) network. They constructed a composite hydrogel with tunable upper critical solution temperature (UCST) and lower critical solution temperature (LCST). The introduction of CMC not only increased the phase transition temperature but also greatly improved the mechanical stability of the hydrogel. Improving the molecular structure can effectively enhance its responsiveness. However, being a molecular-level modification, it has high costs, limited operational space, and uncertainty in grafting. Therefore, more precise research methods are needed.

Introducing specific functional fillers into hydrogels can not only improve the mechanical properties but also act as a “catalyst” to enhance the speed and extent of temperature response. The most commonly used functional fillers include β-cyclodextrin, magnetic Fe_3_O_4_, and CDs. Wu et al. [69] used a one-pot method in a NaOH/urea/water system. They grafted β-cyclodextrin onto cellulose using it as a filler. They also utilized the host–guest interaction between β-cyclodextrin and polypropylene glycol (PPG) to construct a supramolecular hydrogel. The LCST of this hydrogel was 34 °C. At temperatures below the LCST, water molecules penetrate, making the supramolecular hydrogel network looser. It has a high equilibrium swelling ratio, which is more conducive to drug release [70]. When the temperature exceeds the LCST, hydrogen bonds between water molecules and β-CD are broken. The hydrophobic interaction between β-CD and PPG is further enhanced, causing the hydrogel network to contract, and the equilibrium swelling ratio decreases rapidly. It has good network structural stability and thermal stability, showing great potential in intelligent responsive applications [71]. Recently, Xu et al. [72] modified TEMPO-oxidized cellulose nanofibers with Fe_3_O_4_, successfully introducing magnetic materials into the system. Fe_3_O_4_ nanomagnetic particles can generate heat under an alternating magnetic field. This locally raises the hydrogel temperature, triggering the phase transition of the thermosensitive component. Carbon dots (CDs) also have good thermal conductivity and biocompatibility. Adding them to thermosensitive hydrogels can transfer heat to the entire network more quickly and uniformly. This accelerates the phase transition process of the thermosensitive component and improves response speed. Recently, graphene quantum dots, due to their good thermal conductivity, can enable thermosensitive polymers like PNIPAM to respond to temperature changes more uniformly and rapidly. Shi et al. [73] used PNIPAM as the substrate. They introduced carbonized polymer dots prepared by a hydrothermal method from dopamine-grafted dialdehyde cellulose (DAC) into the hydrogel network. This not only weakened the polymerization inhibition feature of phenolic hydroxyl groups but also effectively used carbon dots to improve the temperature response capability of the hydrogel. Although adding functional fillers is relatively simple and efficient, it improves the temperature response performance and can comprehensively enhance various properties like mechanical, conductive, and thermal properties, even introducing new stimulus responsiveness (e.g., magnetic response) for multifunctionality, and the biocompatibility, dispersion stability, and potential long-term toxicity of introduced fillers remain issues that need current focus.

**Figure 6:**
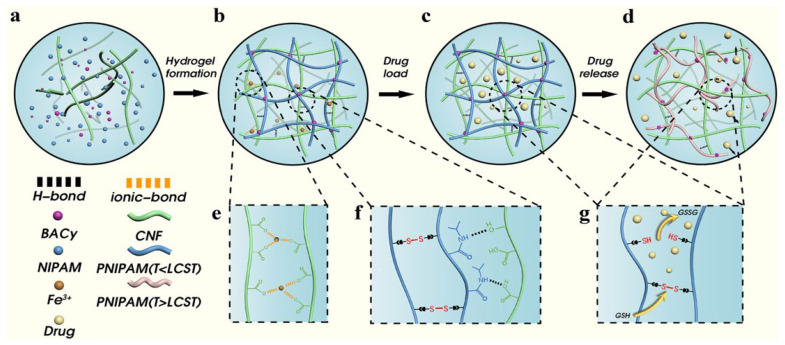
The hydrogel formation and responsive release mechanism of hydrogels ((**a**) Raw materials for preparation of hydrogels. (**b**) CNF/NIPAM hydrogel forms network structure through cross-linking. (**c**) Drug loading in hydrogels. (**d**) Drug release process of hydrogel. (**e**) Ionic bond between Fe^3+^ and carboxyl group on CNF. (**f**) PNIPAM are connected by BACy; NIPAM and CNF are connected by hydrogen bond. (**g**) Disulfide bond breaking and drug release under reductive conditions.). Reproduced with permission from Ref. [48]. Copyright 2022, Elsevier.

Constructing a dual-network structure involves building interpenetrating or semi-interpenetrating networks. This allows the thermosensitive polymer network and the cellulose network to interpenetrate and effectively solves problems like slow response or poor mechanical performance in a single network. Zong et al. [48] designed the introduction of a physically cross-linked network composed of TEMPO-oxidized cellulose nanofibers (CNFs) as the first network (Figure 6). This enhanced the compressive resistance and mechanical properties of the hydrogel. They used PNIPAM as the second network, responsible for temperature response. It undergoes a significant volume phase transition near its LCST (about 32–34 °C), thereby controlling drug release. Furthermore, Li et al. [49] used CMC as the first network and a copolymer of PNIPAM and AA [P(NIPAM-co-AA)] as the second network. The PNIPAM segments are responsible for the temperature response. The PAA segments, whose ionization degree changes with pH, give the hydrogel pH responsiveness. This forms a temperature-pH dual response system. It enables the hydrogel to achieve intelligent drug release targeted at specific pathological environments. Drug loading experiments proved that the release rate of the model drug 5-fluorouracil was significantly higher at 40 °C and under acidic conditions than at room temperature and neutral conditions. Gong et al. [74] used cellulose-based carbon dots derived from lychee waste as a cross-linker. They constructed a dual-network hydrogel of β-cyclodextrin/carboxymethyl cellulose/polyvinyl alcohol-poly(*N*-isopropylacrylamide) that possessed both pH and temperature dual response characteristics. The LCST of this hydrogel was close to physiological temperature. Meanwhile, the pH responsiveness of the hydrogel also caused differentiated swelling behavior and drug release rates under different pH environments. The construction of dual networks cleverly combines the excellent mechanical properties of cellulose with the sensitive response of thermosensitive polymers. It achieves a significant “1 + 1 > 2” effect, suitable for drug delivery occasions requiring certain pressure resistance. However, the optimal design of the network structure, the compatibility of the two polymers, and the complexity of the preparation process are challenges that cannot be ignored.

## 4. Applications of Cellulose-Based Hydrogels in Drug Delivery

Cellulose is known for its biocompatibility, low production cost, abundant sources, and non-toxic nature. Hydrogels prepared primarily from cellulose are now widely used in biomedicine. From serving as drug carriers to ensure that drugs reach the target site to enabling precise drug release, and finally aiding in biological regulation for active healing, cellulose-based hydrogels have a promising future in this field.

### 4.1. Drug Carrier

Cellulose-based hydrogels offer multiple advantages as drug carriers. These include high specific surface area, tunable pore structure, and good biocompatibility. Researchers have designed various cellulose-based hydrogel carrier systems tailored to different administration routes, such as oral, transdermal, and injection. This enables efficient drug loading and targeted delivery. As shown in Table 6, medical researchers choose the appropriate administration method based on specific needs.

Oral administration is the most common drug delivery method. Drugs are absorbed into the bloodstream through the gastrointestinal tract and then distributed throughout the body to achieve therapeutic effects. This method is more acceptable to patients compared to subcutaneous or injectable routes. However, the gastrointestinal environment is unique. Some drugs lose their activity before reaching the target site, resulting in low utilization rates. The human body lacks cellulase enzymes. Therefore, cellulose-based hydrogels can protect drugs from the harsh GI environment. They enable controlled release at specific sites, improving drug utilization. Liu et al. [90] prepared mesoporous polydopamine nanoparticles to load tetracycline hydrochloride. These were then wrapped with graphene oxide and encapsulated within a CNF hydrogel to form a composite material. This double encapsulation significantly reduced the initial burst release of the drug and also extended the sustained release time. It shielded the drug from GI interference, allowing it to reach the target site effectively. Ghawanmeh et al. [75] used 5-fluorouracil as a model drug and optimized the ratio of CMC and gum Arabic using response surface methodology. These prepared hydrogel beads are for anticancer drug delivery. Studies proved that when CMC = 99.61 mg and GA = 77.84 mg, the drug encapsulation efficiency of the hydrogel beads reached 55.7%. Won et al. [76] found that CNF-based hydrogels can protect drugs from strong acids and digestive enzymes. The release rates of IBU and SDS varied at different pH values. The maximum swelling rate of the SDS hydrogel reached its peak at the 24th hour. In the acidic environment of the stomach and duodenum, these carboxyl groups are protonated, and the polymer chains contract, encapsulating the drug within. When it reaches the neutral pH environment of the colon, the carboxyl groups ionize, generating a strong electrostatic repulsion, causing the hydrogel to rapidly swell and release the drug. If stimulus-responsive elements are introduced, it can further achieve the controlled release of drugs at specific intestinal sites (such as the ileum or colon) [77]. Although laboratory research achievements are plentiful, oral delivery systems based on nanocellulose hydrogels still face many challenges in clinical translation and large-scale application. Future oral drug delivery may develop toward more precise and intelligent controlled release.

**Figure 7:**
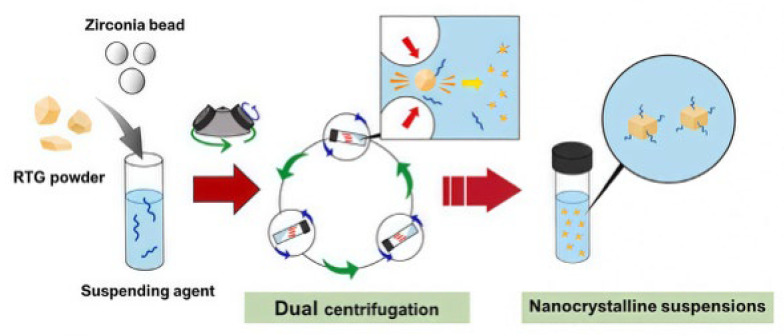
Illustration of the fabrication process of RTG-loaded NSs using the dual centrifugation technique. Reproduced with permission from Ref. [79]. Copyright 2024, Elsevier.

Transdermal administration delivers drugs through the skin for local or systemic treatment. It avoids first-pass liver metabolism and degradation by stomach acid. Early on, Zsikó et al. [78] studied incorporating drug nanocrystal suspensions into polymer hydrogels for skin delivery. This provided a high drug concentration gradient for skin absorption. Park et al. [79] used a dual-centrifugation technique (Figure 7) and successfully constructed a drug NS-loaded hydrogel using polysaccharides as both a suspending agent and hydrogel matrix. Cellulose derivatives uniformly disperse and immobilize drug nanocrystals in the hydrogel matrix through electrostatic interactions. This enables transdermal delivery and reduces skin irritation caused by the drug. It offers better treatment for Parkinson’s disease. Recently, Yang et al. [80] developed a composite hydrogel dressing for wound treatment. This dressing incorporated berberine-loaded silk fibroin microspheres and calotropis gigantea fiber into a sodium alginate hydrogel matrix and achieved the long-term release of berberine. The dressing also showed significant inhibitory effects on Staphylococcus aureus. It addresses limitations like rapid drug release and insufficient mechanical strength in hydrogel dressings. It also has a hemostatic effect. Although transdermal delivery reduces the liver’s burden, enables local, precise treatment, is convenient to operate, and is suitable for long-term chronic therapy, its manufacturing process is complex. It is incapable of delivering large molecular particles, and some carrier materials can cause skin irritation. Therefore, it is not suitable for all drugs.

**Figure 8:**
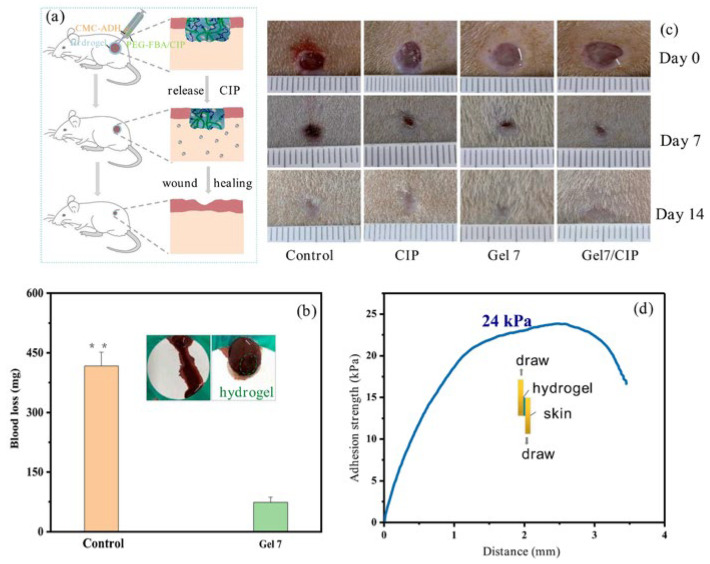
(**a**) Illustration of accelerated wound-healing of CMC-ADH/PEG-FBA hydrogels. (**b**) Hemostatic performance of the CMC-ADH/PEG-FBA hydrogel (Gel7), the value of * = 416.7 mg; inset: pictures of bleeding mouse liver; (**c**) pictures of wounds at 0th, 7th, and 14th day for Tegaderm film dressing (control), CIP, Gel7, Gel7/CIP. (**d**) The adhesion strength of the hydrogel to skin tissue. Reproduced with permission from Ref. [82]. Copyright 2023, Elsevier.

Parenteral administration involves directly introducing drugs into the body for rapid absorption and effect. In the field of cellulose-based hydrogel drug delivery, it typically means that the drug is loaded within a hydrogel carrier. This is then injected into a specific site to prolong drug release time and reduce administration frequency. As early as 1996, Mi et al. [81] designed a method involving solidification in liquid. They prepared CM-chitin microspheres for the sustained release of anticancer agents. Using 6-mercaptopurine as a reference core, they incorporated it into the gel after ion cross-linking with ferric chloride. The drug release rate of the resulting product decreased with increasing Fe^3+^ concentration and curing time. Li et al. [82] synthesized a CMC-ADH/PEG-FBA hydrogel. Experiments proved it had good injectability. This could be used for wound treatment in mouse models of internal bleeding and full-thickness skin defects. As shown in Figure 8b, compared to the control group, mice treated with the gel dressing showed significantly reduced bleeding. It not only promoted the wound healing process but also exhibited an excellent hemostatic effect. Figure 8d shows the strong adhesion strength of the hydrogel to skin tissue, which is more conducive to achieving hemostasis. Recently, Zhou et al. [83] proposed an injectable dual-drug delivery system based on a cellulose-derived microgel/hydrogel composite. This system showed strong potential for selectively targeting gastric cancer cells. It minimized cytotoxicity to healthy tissues. This delivery strategy enables precise intratumoral drug delivery. It also reduces systemic toxicity and surgical complexity. It can be used for coordinated dual-drug delivery. Injectable administration allows drugs to enter the bloodstream directly through veins, acting quickly. For some special patients, such as those in a coma, with intestinal obstruction, or difficulty swallowing, injection may be the only route. However, due to the high pain level, it creates invasive wounds and carries a risk of infection. Therefore, achieving low-cost, efficient sterilization and introducing needle-free injection technology have become current challenges.

Ocular administration is a localized drug delivery method that involves applying medications directly to the eye surface. That is what we commonly refer to as eye drops. A major challenge in this approach is the eye’s highly efficient self-protection mechanisms, such as the blink reflex and tear washout. These mechanisms rapidly remove instilled drugs, resulting in an intraocular bioavailability typically below 5% [84,85,91]. Hydrogel-based matrices offer a promising solution due to their excellent mucoadhesive properties. They significantly prolong the residence time of the formulation on the corneal surface. Moreover, the three-dimensional network structure of hydrogels enables diffusion-controlled drug release. This helps maintain effective drug concentrations on the ocular surface and reduces the dosing frequency. Consequently, drug permeation across the cornea and therapeutic efficacy are significantly enhanced. As early as 2015, Nibourg et al. [91] developed a nanofiber-based nanogel to serve as an extracellular environment for lens epithelial cells (LECs). They used a porcine eye model to evaluate the effect on posterior capsule opacification (PCO) after lens surgery. The extracted lenses were cultured for three weeks. Compared with the control group filled with hyaluronic acid, the nanogel helped maintain the normal epithelial-like morphology of LECs. It also reduced the expression of α-smooth muscle actin, thereby inhibiting PCO formation. Furthermore, results indicated that the nanogel enhanced cell adhesion, offering a potential advantage for ocular drug delivery. In 2021, Lin et al. [85] developed an ocular in situ gelling drug delivery system based on Poloxamer 407. This system exhibits thermoreversible gelation, high water solubility, and the ability to form a transparent gel. These properties make it suitable for widespread use in ocular drug delivery. It addresses the limitations of conventional eye solutions, which often show poor therapeutic efficacy. The system enhances drug penetration, improves therapeutic effects, and reduces the drug release rate. Nagai et al. [86] successfully developed an ophthalmic in situ gelling (ISG) formulation using TL-NPs. Their research demonstrated that the in situ gelling system, based on methylcellulose (MC) and TL-NPs, extended the ocular residence time. It also enhanced drug uptake in both the cornea and conjunctiva. These findings underscore the considerable potential of methylcellulose for ocular delivery platforms. Although cellulose-based hydrogels have significantly improved the efficiency of ocular drug delivery, several challenges remain. These include limited drug-loading capacity, insufficient precise control over drug release behavior, and the need for the further validation of long-term biocompatibility. Future research should focus on developing intelligent responsive systems. Expanding their application to macromolecular drug delivery, such as gene therapeutics, is also essential.

Nasal drug delivery offers a non-invasive route of administration. Its significance lies not only in treating local nasal disorders but also in enabling rapid systemic drug absorption through the highly vascularized nasal mucosa. This route avoids first-pass metabolism and bypasses the blood–brain barrier, thereby improving patient compliance. It also presents new opportunities for treating central nervous system diseases. However, a major challenge associated with this method is the rapid clearance caused by nasal cilia, which considerably shortens the drug absorption window. Gonzále et al. [87] investigated hydroxypropyl methylcellulose (HPMC), a polymer commonly used for release control in hydrophilic matrix tablets. Their study demonstrated that an increase in HPMC particle size generally leads to a higher drug release rate from matrix tablets. In contrast, higher viscosity grades were found to slow down the release rate. Therefore, by tailoring the particle size of HPMC, the drug release profile can be modulated. This approach enables sustained drug release in the nasal cavity. In 2022, Kesavan et al. [88] developed a mucoadhesive microemulsion for the nasal delivery of dexamethasone. Carboxymethyl cellulose was employed as a viscosity-enhancing agent. It acted synergistically with chitosan to form a stronger mucoadhesive system. This system was designed to prolong the ocular residence time of drugs. It is also suitable for nasal drug delivery, thereby improving drug utilization. In 2024, Hosseini et al. [89] designed a novel immunomodulatory wound dressing based on CMC and alginate. This hydrogel demonstrated good biocompatibility and muco-adhesive properties. It can anchor formulations to the nasal mucosa, effectively countering clearance mechanisms. Consequently, it creates a longer window for drug absorption. The dressing also exhibits potential for enhancing and accelerating skin regeneration, which is beneficial for managing chronic wound healing. Although material innovations have successfully prolonged the nasal drug residence time, several challenges remain in this field. These include limited drug loading efficiency, long-term mucosal safety concerns, and significant inter-individual variability in drug absorption. Future research needs to address these issues. Efforts should focus on developing more biocompatible, smart-responsive hydrogel systems. Additionally, exploring their clinical potential for nose-to-brain delivery of macromolecular drugs, such as nucleic acids and proteins, is absolutely necessary.

### 4.2. Drug Release

The precise release of drugs from hydrogels at specific sites involves stimulus responsiveness. In recent years, researchers have developed various stimulus-responsive hydrogels. These include pH-responsive, photothermal-responsive, and multi-responsive systems. As shown in Table 7, they can control drug release behavior based on environmental changes. This enables drug release at a specific time, location, and predetermined rate, therefore improving the therapeutic efficacy while reducing side effects.

**Figure 9:**
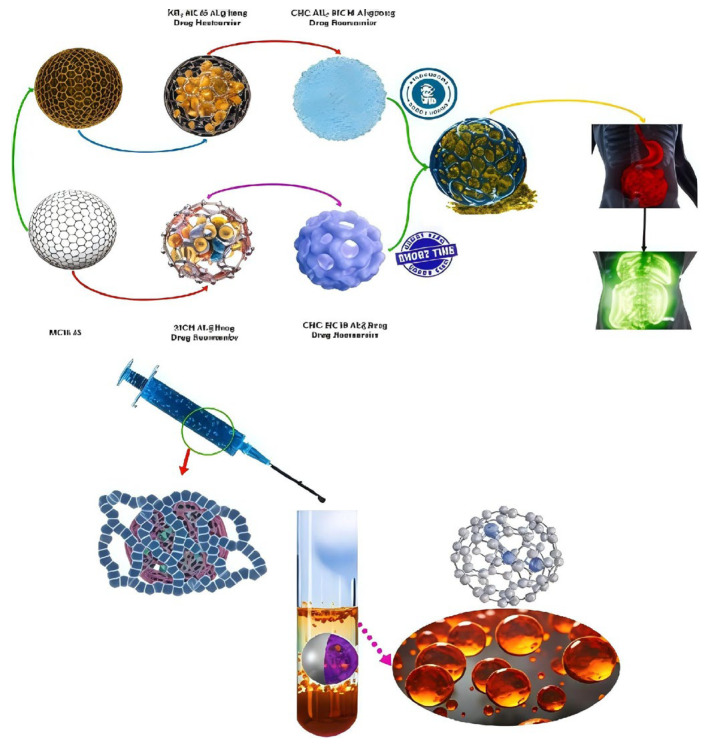
Schematic of the bead preparation of hydrogel drug nanocarriers. Reproduced with permission from Ref. [93]. Copyright 2024, Elsevier.

pH-responsive hydrogels are one of the most widely studied drug release systems. The gel network swells or shrinks at different pH levels, achieving controlled drug release. In this regard, carboxymethyl cellulose shows great potential due to its excellent pH responsiveness. Different human organs, tissues, and diseased areas have varying pH levels. The tumor microenvironment is weakly acidic, gastritis areas are highly acidic, and the intestinal environment is alkaline. This provides a basis for precise drug release applications. Li et al. [82] formed an inner layer by cross-linking SA and CMC with Ca^2+^. The outer layer was prepared from acrylamide derivatives via free radical polymerization. This avoided premature drug leakage and burst release in the stomach. It also enhanced the system’s strength and stability. Drug loading experiments proved that the sustained release effect for different model drugs could be adjusted by changing the composition or thickness of the hydrogel layers. This achieved precise release in the intestinal environment. Pooresmaeil et al. [100] used dialdehyde-modified CMC to crosslink nano-gels. This achieved targeted drug delivery for breast cancer treatment. Experiments proved that due to changes in pH, the rupture of the nanogels, weakened hydrogen bonds, and π–π stacking interactions, etc., the gel swelling coefficient changes, and the drug release begins. It showed minimal drug release at neutral or gastric pH, but release increased at weakly acidic pH. Shirazian et al. [93] modified CMC to prepare the nanocomposite hydrogel as shown in Figure 9, which was loaded with drugs. In vitro release results showed similar drug loading results for doxorubicin, naproxen, and metformin. The drug release rate was much lower in SGF compared to SIF and SBF. The synthesized hydrogels open new horizons for pH-sensitive drug carriers. They offer slow, controlled release rates and optimal drug loading capacity. Recently, Xia et al. [94] developed a pH-responsive and colon-targeted delivery system by encapsulating self-assembled BBR-RA nanoparticles (BBR-RA NPs) within Ca^2+^-crosslinked alginate (ALG)/sodium carboxymethyl cellulose (CMC) hydrogels (ALG/CMC/BBR-RA NPs) for colon-targeted delivery. Among them, CMC provided pH-responsive capabilities. This gel has low swelling in the acidic gastric environment, protecting the drug. Upon entering the neutral or weakly alkaline colon environment, the gel swells and gradually degrades, achieving targeted drug release. In vitro release experiments confirmed that this system effectively increased drug uptake in colon cancer cells, ROS generation, and apoptosis. However, biological models are still needed to further verify their biosafety. Although the carboxyl groups in CMC provide pH responsiveness, good biocompatibility, and a clear response mechanism enabling targeted release, its mechanical strength is weak. Its loading efficiency for hydrophobic drugs is low. Future development may focus on constructing multiple response systems, improving drug selectivity, and enhancing clinical applicability.

Photothermal response involves special materials in the hydrogel that efficiently convert light energy into heat upon exposure to specific wavelengths. Near-infrared light is often used due to good tissue penetration and low absorption. Red light penetrates skin more deeply. In drug delivery systems, this localized heat can trigger physical or chemical changes in the hydrogel network. This enables on-demand, controlled release of the carried drug. DOX is a common drug for cancer treatment. Chang et al. [95] used CMC-CHO and PNH as raw materials and combined them with the NIR-triggered photothermal properties of GNRs. They successfully prepared a biodegradable, self-healing hydrogel with thermal response. Drug loading experiments proved the hydrogel’s phase transition could be adjusted near body temperature. The hydrogel exhibited different DOX release profiles below and above the LCST. Combining the sustained release performance of the hydrogel with the advantages of NIR photothermal-triggered phase transition, this GNR composite hydrogel has great application potential in bioscience and biotechnology. Cheng et al. [101] used a PVA-borax/GelMA hybrid hydrogel as the matrix to prepare a PGTMO hydrogel. BC enhanced the mechanical properties and acid (TA)-modified MXene nanosheets, which imparted superior antibacterial efficacy. Experiments proved that its photothermal conversion efficiency was 46.5% under NIR irradiation. It also showed an over 95% inhibition rate against drug-resistant bacteria. Applying this gel in medicine not only enables NIR-triggered on-demand DOX release, but also synergizes with photothermal therapy to kill tumor cells. It shows high potential in treating tumors with accompanying infections or preventing post-operative recurrence. Wang et al. [96] compounded cellulose extracted from bulk agricultural waste corn cobs with carbon nanotubes. This produced CNT/cellulose hydrogel composites. The efficient photothermal conversion capability of CNTs induces changes in the hydrogel network structure. This provides a new idea for scientific research. One could try adding CNTs to cellulose-based hydrogels for the NIR-triggered release of drugs like DOX. This could attempt remote treatment while effectively reducing the material costs. Although photothermal-responsive cellulose hydrogels show great advantages in DOX delivery, the long in vivo metabolic cycle and potential toxicity of many nano-photothermal materials remain a major challenge. Also, NIR’s tissue penetration ability is limited, potentially restricting its effect on deep tumors. Future efforts should focus on finding biodegradable heat-conducting materials and constructing multiple response systems.

The mechanism of enzyme-responsive hydrogels is based on enzymatic reactions that disrupt or modify the hydrogel network. Researchers selectively introduce specific substrates into the hydrogel’s cross-links or polymer backbone. These substrates can be recognized and cleaved by specific enzymes. When the hydrogel is in an environment containing the target enzyme, the enzyme recognizes and binds to the substrate, catalyzing its hydrolysis. This destroys the hydrogel structure, causing a sharp increase in network swelling or degradation, thereby releasing the encapsulated drug and achieving targeted release. Especially for the colon region, where there are various characteristic bacterial colonies. Bao et al. [97] designed cellulose-based nanomaterials to enter tumor regions via the enhanced permeability and retention effect and ligand-mediated active targeting mechanisms. Multiple enzymes are overexpressed in the tumor microenvironment. These enzymes can specifically cleave peptide cross-links in the hydrogel, triggering drug release. Therefore, the presence of these enzymes can enhance the delivery efficiency of chemotherapeutic drugs. They also show potential in overcoming multidrug resistance and reducing systemic toxicity. Wu et al. [98] cleverly utilized the overexpression of three enzymes in cancer cells: phosphatase, esterase, and protease. The lonidamine-peptide conjugate serves as both a drug precursor and carrier. Inside cancer cells, ALP and CES sequentially catalyze this molecule, causing its intracellular self-assembly into nanofiber hydrogels. This protects the drug from premature release. Normal cells have low enzyme content and cannot effectively activate the release system. Subsequently, cellular proteases further hydrolyze the hydrogel, controlling the slow release of lonidamine. Recently, Gao et al. [99] grafted CMC onto hollow mesoporous silica nanoparticles and loaded the fungicide hexaconazole. When pathogenic fungi infect, they secrete cellulase and organic acids, destroying the carrier structure. This achieves precise drug release at the infection site. This system significantly improved efficacy while reducing environmental residue. Although applied to rice sheath blight disease, it opens a new avenue. We can design intelligent antimicrobial delivery systems targeting specific secreted enzymes by adding specific raw materials. Cellulose-based enzyme-responsive hydrogels hold great potential in tumor-targeted therapy, inflammatory disease treatment, and certain fungal infection therapies. However, the in vivo environment is complex and variable. We must ensure that the hydrogel responds only to specific target enzymes, not premature release. The long-term stability and clinical safety of hydrogels also need consideration. Future research may focus more on developing multiple response systems. Using synthetic biology techniques to more precisely customize cellulose materials or corresponding enzymes could achieve more accurate, efficient, and safe drug delivery.

### 4.3. Wound Dressings

Cellulose-based hydrogels create a moist healing environment through their three-dimensional network structure. They can absorb large amounts of exudate and lock in moisture, preventing wound dryness and scabbing. This significantly accelerates epithelial cell migration and wound healing. Simultaneously, they allow for oxygen permeation and effectively block external bacteria, making them excellent materials for wound dressings. They have excellent biocompatibility and do not adhere to new granulation tissue. This can greatly reduce the pain of dressing changes. Furthermore, they can serve as intelligent drug carriers, loading and controlling the release of antibiotics, growth factors, and other drugs. This integrates anti-infection and pro-healing functions. They are an ideal platform for new functional dressings. As shown in Table 8, wound dressings also have their corresponding application scenarios.

**Figure 10:**
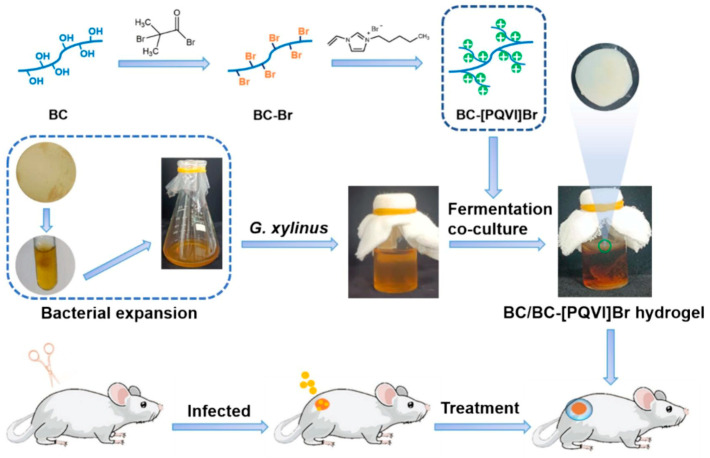
The schematic diagram of BC/BC-[PQVI]Br hydrogel dressing and the treatment of wound infection in vivo (stirred at room temperature for 24 h). Reproduced with permission from Ref. [104]. Copyright 2024, Elsevier.

Hosseini et al. [102] developed a hydrogel formed from methacrylated cellulose and a chitosan derivative. It created an interpenetrating polymer network and could rapidly cure under light. It effectively prevented E. coli biofilm formation without any antibiotics. It exhibited excellent anti-fouling performance and blood compatibility. It is a potential substitute for traditional gauze dressings. Wang et al. [103] combined the biocompatibility of bacterial cellulose with the hemostatic function of a recombinant thrombin-cellulose binding domain fusion protein. They made a T-BC dressing that achieved rapid hemostasis in a rat liver model within one minute. It also accelerated wound repair. It showed great potential in treating deep burn wounds. Meng et al. [104] designed a BC-based hydrogel with inherent antibacterial function (Figure 10). Its maximum tensile stress reached 970 kPa and could moisturize for 6 h. Its antibacterial rates against Staphylococcus aureus and Escherichia coli were 93% and 71%, respectively. The cell survival rate was 98%. Mouse wound experiments confirmed its good healing effect. It could serve as an excellent dressing. However, the current application is limited to clinical mice, requiring further study. Overall, cellulose-based hydrogel dressings show great promise for replacing traditional dressings. This is due to their excellent biocompatibility, high fluid absorption and moisture retention capacity, and the core advantage of providing a moist wound healing environment. Their potential as a multifunctional drug delivery platform also contributes. However, their weak mechanical properties, limited loading efficiency for hydrophobic drugs, long-term stability, and large-scale production costs remain major challenges. Future research will focus on developing intelligent responsive hydrogels integrating multiple functions like antibacterial, anti-inflammatory, and pro-angiogenic. It will also promote their development toward theranostics and clinical translation. The ultimate goal is the precise and efficient management of complex situations like chronic hard-to-heal wounds.

## 5. Conclusions and Prospects

This review has comprehensively examined the preparation methods, modification strategies, and applications of cellulose-based hydrogels in drug delivery. The unique structure of cellulose underpins their versatile properties: abundant hydroxyl groups enable precise chemical modification, allowing for the introduction of stimuli-responsive moieties to control drug release. The inherent three-dimensional porous network provides an ideal matrix for drug loading, while their tunable mechanical properties support diverse applications, from injectable systems to wound dressings. Furthermore, their excellent biocompatibility and biodegradability ensure fundamental biosafety. These characteristics collectively highlight the significant potential of cellulose-based hydrogels. Nevertheless, several challenges remain and warrant further investigation:

(1) Nevertheless, in the field of hydrogel preparation, the strong hydrogen bonding network and high crystallinity of cellulose, coupled with the frequent use of toxic crosslinking agents, often conflict with green chemistry principles, despite the diversity of existing methods. Future research should be directed toward fully leveraging cellulose’s inherent advantages—such as its renewability and biodegradability—while developing novel green solvent systems. This will help minimize the use of hazardous reagents and enable more energy-efficient and environmentally benign synthesis routes. Moreover, advancing fabrication processes from laboratory-scale to large-scale, sterile production that complies with medical device standards is considered essential for successful clinical translation.
(2) Regarding hydrogel modification, the inherent lack of physiologically responsive functional groups in cellulose, combined with the structural incompatibility often encountered when constructing dual-network architectures, hinders the achievement of synergistic reinforcement effects. To overcome the trade-off between mechanical properties and stimulus responsiveness, future strategies should involve more precise designs of double-network or interpenetrating network structures as well as the introduction of dynamic covalent bonds. These approaches can endow hydrogels with self-healing, remoldable, and stimulus-responsive capabilities in complex physiological environments. Deep integration with artificial intelligence, particularly through machine learning algorithms, can facilitate the analysis of complex nonlinear relationships among modifier types, crosslinking density, reaction conditions, and final performance metrics (e.g., strength, modulus, swelling ratio). This would enable the rapid screening of optimal modification formulations. Furthermore, AI-driven approaches can support the reverse design of hydrogel chemical structures based on specific biological signals at the target site (e.g., enzyme concentration, pH range), thereby laying a chemical foundation for precise drug delivery.
(3) In terms of drug delivery, future systems must not only ensure biosafety, but also prioritize stimulus responsiveness as a core design element. 3D/4D printing technologies offer powerful platforms for achieving this goal. 3D printing allows for precise control over the macroscopic structure and internal porosity of hydrogel carriers, enabling the fabrication of drug formulations with specific geometries and drug distribution gradients. 4D printing introduces an additional “time” dimension, where printed cellulose-based hydrogel structures can undergo programmed shape changes, swelling, or degradation in response to predefined stimuli in the body (e.g., body temperature, inflammatory pH). Through such advances, cellulose-based hydrogels are expected to evolve beyond their current role as simple carriers into intelligent medical systems capable of sensing, decision-making, and therapeutic execution.

## Figures and Tables

**Table 1:** Comparison of different preparation methods of cellulose-based hydrogels.

Preparation Method	Key Advantages	Main Limitations	Application Fields	Reference
Physical Cross-linking	No toxicity residue; Good bio-compatibility; Low cost	Low mechanical strength; Stability easily affected by calcium ion concentration; Low mechanical strength	Drug delivery; Water adsorption; Drug delivery; Water adsorption	[10,11,12,13]
Chemical Cross-linking	High mechanical strength; Excellent stability; Strong anti-swelling ability	Irreversible reaction; Cross-linker residue, possible biological toxicity	Long-acting drug controlled release; Extreme environments	[14,15,16,17]
Bio-dynamic Cross-linking	Simulate biological mechanism; Self-adaptive ability; Excellent bio-compatibility	Difficult multi-scale synergistic cross-linking	Wound repair dressings	[18,19,20]
Photo-initiated Cross-linking	Precise cross-linking sites; Second-level curing speed; Low cost	Some photoinitiators have cytotoxicity	Wound repair dressings	[21,22,23]

**Table 2:** Comparison of Different Methods in Physical Crosslinking Technique.

Preparation Method	Reaction Principle	Main Limitations	Application Fields	Reference
Hydrogen bond dynamic cross-linking	Use reversible hydrogen bonds between cellulose chains to create a 3D network	Insufficient mechanical strength; Easily disturbed in the physiological environment	Drug-controlled-release carriers; Intelligent responsive sensors	[10,11,25]
Ionic dynamic cross-linking method	The ionic interactions form the 3D network structure of the hydrogel	Low mechanical strength; Poor stability at high ion concentrations	Drug-controlled-release carriers; Wound dressing	[12,26,27]
Electrostatic interactions	Form a 3D network through electrostatic attraction between oppositely charged polyelectrolytes	Poor mechanical stability; Poor drug-loading capacity	Drug-controlled-release carriers; Soft tissue filler	[13,28,29]

**Table 3:** Comparison of Different Methods in Chemical Crosslinking Technique.

Preparation Method	Reaction Principle	Main Limitations	Application Fields	Reference
Covalent Cross-Linking	Form a three-dimensional network structure through irreversible chemical bonds	Irreversible reaction; Residual toxicity of crosslinking agent	Drug-controlled-release carriers; Soft tissue filler	[14,34,35]
Schiff Base Reaction	Condensation of an aldehyde or ketone with a primary or secondary amine under acidic or neutral conditions	Complex synthesis process; Poor long-term stability	Injectable nanogels; Biosensors for blood glucose detection	[15,36,37]
Esterification Reaction	The hydroxyl groups react with crosslinking agents such as acid anhydrides to form ester bonds	Uneven replacement degree; Residual reagents	Drug-controlled-release carriers; Injectable nanogels	[16,17,38]

**Table 4:** Comparison of other preparation methods.

Preparation Method	Reaction Principle	Main Limitations	Application Fields	Reference
Bio-dynamic Cross-linking	Mimics reaction mechanisms between biological molecules within a biomaterial system	Highly targeted; Complex preparation process	Treatment of diabetes; Early burn management	[18,19,20]
Photo-initiated Cross-Linking	Light initiates the generation of reactive free radicals, triggering the polymerization of monomers	Some photoinitiators have toxicity; Limited ability to penetrate the skin through light	Wound healing; Rapid hemostasis during surgery	[21,22,23]

**Table 5:** Comparison of different types of functional modification of cellulose-based hydrogels.

Modification Type	Key Advantages	Main Limitations	Reference
Self-healing Performance	Prolong service life; As an injection carrier, it can reduce invasive surgery	Insufficient stability; Complex production process; Bio-compatibility affected by metal fillers	[41,42,43]
PH Responsiveness	Realize targeted drug release; Increase drug loading capacity	Response consistency is easily affected; Difficult to scale production; High cost	[44,45,46]
Temperature Responsiveness	Rapid response; High mechanical strength of double-network structure; Adapt to complex physiological environments	High cost of thermosensitive polymer grafting; Unstable production efficiency; Poor polymer compatibility	[47,48,49]

**Table 6:** Comparison of Cellulose-Based Hydrogel Drug Carriers in Practical Applications.

Application Route	Core Function	Key Advantages	Main Challenges	Reference
Oral Administration	Gastrointestinal-protective & colon-targeted drug release	High patient compliance; Improved drug utilization; Improved drug utilization	Nanocellulose system clinical translation difficulty; Large-scale production process; translation difficulty	[75,76,77]
Transdermal Administration	Liver/gastrointestinal degradation avoidance; Local/systemic treatment	Convenient operation; Suitability for long-term/chronic treatment; Long-acting drug release; Bacteriostatic/hemostatic effects	Macromolecular drug delivery incapability; Skin irritation of partial carriers	[78,79,80]
Parenteral administration	Rapid drug absorption, prolonged release; Reduced administration frequency; Targeted delivery	Intratumoral precise delivery; Reduced systemic toxicity	Invasive operation-induced infection; Low-cost efficient sterilization; Needle-free injection technology	[81,82,83]
Ocular Administration	Prolonged drug residence time on the ocular surface, enhanced corneal penetration; Targeted delivery to ocular tissues	Improved bioavailability; reduced dosing frequency; patient comfort; suitability for chronic eye diseases	Potential ocular irritation; limited drug permeability due to corneal barriers; sterilization and stability issues	[84,85,86]
Nasal Drug Delivery	Mucoadhesive drug delivery for systemic or local action, bypassing first-pass metabolism	Non-invasive; rapid onset of action; direct delivery to the brain via olfactory pathway; high patient compliance	Rapid clearance by nasal cilia; possible nasal mucosa irritation; variability in absorption	[87,88,89]

**Table 7:** Comparison of Practical Applications of Cellulose-Based Hydrogel in Drug Release.

Response Type	Triggering Mechanism	Targeted Application Scenarios	Key Advantages	Main Challenges	Reference
pH-Responsive	Induced by intra-body pH differences for gel swelling/shrinkage	Tumor-targeted delivery; Intestinal-targeted delivery	Clear response mechanism; Good biocompatibility; Controllable release rate	Weak mechanical strength; loading efficiency	[92,93,94]
Photothermal-Responsive	Photothermal materials convert light to heat for gel phase transition/degradation	Tumor chemotherapy (DOX release); Infected wound photothermal therapy	Remotely precise release control; Integrated photothermal therapy	Potential photothermal material toxicity; Limited NIR penetration for deep tumors	[95,96]
Enzyme-Responsive	Specific enzymes cleave gel crosslinks/main chains to destroy structure	Tumor-targeted release; Fungal infection therapy	Ultra-high targeting; Avoidance of premature drug release; Some systems overcome tumor multidrug resistance	Non-specific responses caused by a complex in vivo environment; Insufficient long-term stability	[97,98,99]

**Table 8:** Comparison of Application Characteristics of Cellulose-Based Hydrogel Wound Dressings.

Dressing Type	Core Function	Key Advantages	Main Challenges	Reference
Antibacterial	Bacteria blocking; Wound infection inhibition; Sterile healing environment	Good biocompatibility; Less dressing-change pain; Antibiotic-free	Weak mechanical strength; Poor long-term stability	[102]
Hemostatic	Rapid wound hemostasis; Coagulation time shortening	High hemostatic efficiency; Wound-conforming without secondary injury	High large-scale production cost; Limited clinical application	[103]
Multifunctional Composite	Integration of moisturizing, antibacterial, hemostatic, drug release functions; Anti-infection-wound healing integration	Comprehensive functions; Complex wound adaptation	Complex material formulation; Difficult preparation process; Low hydrophobic drug loading efficiency	[104]

## Data Availability

No new data were created or analyzed in this study. Data sharing is not applicable to this article.
